# Estimation of Parameters and Pooling in Nonlinear Flooding Event Scenarios with Bayesian Model

**DOI:** 10.1155/2022/6319197

**Published:** 2022-08-28

**Authors:** Fuad S. Alduais, Taghreed M. Jawa

**Affiliations:** ^1^Department of Mathematics, College of Science and Humanities in Al-Kharj, Prince Sattam Bin Abdulaziz University, 11942 Al-Kharj, Saudi Arabia; ^2^Department of Mathematics and Statistics, College of Sciences, P.O. Box 11099, Taif University, Taif 21944, Saudi Arabia

## Abstract

Water-related tragedies are the highest common of all proven natural calamities and pose severe attacks on people and on socioeconomic status development. Due to the obvious controversy surrounding, their volume, location and time of incidence, geological swaths, and geophysical interrelations, flood events are difficult to completely control. Hence, complete flood prevention is always considered to be a viable choice. The specialized flood occurrences are investigated by developing a structural measure. In this paper, nonlinear flood event circumstance is determined by using a statistical Bayesian parametric approach for parameter estimation. A popular tool for estimating a flood design is model of nonlinear flood event. Nonlinear flood event models are subjected to a Bayesian technique for estimating parameter. The approach is based on the minimization function of square for models with nonlinear calculated peak discharges in terms of parameters. The observed and calculated peak discharges for numerous storms in the watershed, data on the pattern of error observed, and previous information on values of parameter all influence this objective function. The subsequent matrix for covariance is a measure of the calculated parameters' accuracy. Rainfall and runoff data from a Harvey River sample are used in this study to show the process.

## 1. Introduction

Water-related disasters are the most prevalent of all natural disasters documented, posing major threats to people and socioeconomic growth. Between 1900 and 2006, floods were responsible for more than 30% of all natural disasters, killing more than 19% of all people and harming more than 48% of the population [1]. Floods account for 26% of all natural catastrophe expenses, with water-related calamities accounting for 72% of all costs. Change in climate, land use changes, sea level increase, deforestation, and development of population in flood-prone areas are all expected to worsen these losses in the future, pushing the global flood disaster population to two billion people [2].

For a number of reasons, optimal forecasting of flood and sustainable risk management systems for flood have been promoted as flood preparation strategies. Floods are difficult to entirely regulate because of the uncertainties surrounding their volume, time, and location of occurrence, geographical expanse, and geophysical interactions. As a result, total flood protection is not always regarded as a practical option [3]. Traditional flood control, which generally consists of structural protection measures such as dams and levees, focuses on changing the characteristics of a flood to minimize peak heights and geographical extents [4]. Although flood danger is reduced by structural solutions such as dams and embankments, it cannot be entirely eliminated. Furthermore, these interventions are impractical in certain region like remote mountain areas, ineffective for all flood procedures like freezing lake outburst floods, and have negative environmental consequences [5]. Moreover, as seen in the USA, aging flood control infrastructure has significant costs and implications in flood setup that does not provide the desired level of fortification or is vulnerable to collapse. Furthermore, specific flood events are analyzed by designing a structural measure, such as a 1% annual exceeding [6], which is difficult to implement because it is done manually, and increased hydrologic uncertainty has resulted from channel alterations, land use changes, and climate change, making floods less expectable. As a result, structural improvements are almost always doomed to fail [7].

The pooling problem has been solved using sophisticated methods. A weighted linear combination of parameter estimations is used in the bootstrapped estimates. The validity of pooling is explicitly assumed in this procedure [8]. Parameter variation is represented in a Bayesian technique by a random parameter model, with storm event parameters chosen from a hyper-distribution that is assumed to be multivariate normal [9]. A variety of models, spanning from fixed to random parameters, can be utilized depending on the model parameterization [10].

Furthermore, there is probable cause to believe that flood event model parameters may be storm-dependent. The model's forecasting power will be harmed unless the parameters have a tight link to easily recognizable elements of the storm (e.g., storm track or rainfall temporal pattern) [11]. The pooling techniques are not useful, but they do presuppose the presence of a universal set of parameters that can be applied to all storms. Individual discrepancies in parameter estimations might be due to a single estimation factor [12].

In NFEMs, manual methods for parameter estimation include trial and error, parameter interaction diagrams, and nonlinear regression modeling [13]. These approaches, with the exception of the method, focus on fitting calculated and pragmatic overflow hydrographs for a single storm. For each storm occurrence, this generates a single set of parameter estimations [14]. When numerous storms are accessible for estimation of parameter, a pooling approach must be used to provide parameter estimation with a single set for the watershed.

The rest of the section is summarized as follows: Section 2 reveals the background of the parameter estimation model; Section 3 details the proposed methodology of Bayesian mechanism in nonlinear flood event detection; Section 5 explains the result and discussion, and finally Section 5 concludes the paper.

## 2. Background

### 2.1. Parameter Estimation and Interference

The calibration of nonlinear flood event is fitted by observed and computed hydrograph peaks with parameter *a* and *m* discrete storm event. The nonlinear flood event model is defined using the regression

(1) $$\begin{array}{c} r_{t}=f\left(i_{t},v\right)+\varepsilon_{t},\;t=1,2,\ldots ,m. \end{array}$$

The input vector is represented as *i*_*t*_ with the observed peak discharge *r*_*t*_, *v* is the unknown parameter with vector, and *F* is the response function which is continuously differential with vector and creates error randomly. The response function (2) of the nonlinear flood event is

(2) $$\begin{array}{c} f\left(i_{t},v\right)=x_{tl}^{\prime },\\ v=\sum_{i=1}^{a}x_{tl}v_{l}, \end{array}$$

where *x*_*tl*_ is component of *i*_*t*_ and transpose matrix of the vector.

The estimation of parameter with statistical approach is based on the literature of hydrologic with least square method. The function of sum of square for estimating the least square with minimum vector is expressed as

(3) $$\begin{array}{c} S\left(v\right)={\left[r-f\left(v\right)\right]}^{\prime }\left[r-f\left(v\right)\right], \end{array}$$

(4) $$\begin{array}{c} S\left(v\right)=\sum_{t=1}^{m}\left[r_{t}-f\left(i_{t},v\right)\right]. \end{array}$$

The column vector and the component of *r* and *f*(*v*) are denoted by *r*_*t*_ and *f*(*i*_*t*_, *v*). The response surface of the contour is denoted by the sum of square function with the linear parameter function for flood event, which is quadratic to the surface and forms hyper-ellipsoid. The unbiased estimation parameter is obtained with the variance, and estimation is based on the interference. The three assumptions should be satisfied for unbiased estimation.

- Specifying the function of model response correctly
- The input vector component is measured without error and is nonstochastic
- The error *ε*_*t*_ obtained is distributed randomly with arbitrary variable normally with mean and constant unknown variance *σ*_*t*_^2^

The unbiased vectors hold the three assumptions with least square, distributed normally, and estimating minimum variance. The preceding properties only hold asymptotically for models with nonlinear parameters. The response surface is nonquadratic for smaller samples, and least squares estimators may be severely skewed and non-normally disseminated, and have variances greater than the smallest feasible variance [15]. The asymmetry of a estimation of least squares for a nonlinear model is the most essential aspect of its distribution. The degree to which this behavior changes depends on the data set combination, and there are no rules on how large a sample must be to approximate one-dimensionality.

### 2.2. Parameter Transformation

Under parameter modifications, the response surface's shape and the sampling characteristics of estimating the least squares are not invariant. It may be feasible to discover a parameterization that produces more quadratic surface response of contours and estimators that approximate the asymptotic requirements. The problem of joint confidence is obtained by simplifying the parameterization for the parameter model.

The parameterization of transformation is obtained alternatively represented by

(5) $$\begin{array}{c} w=t\left(v\right). \end{array}$$

The new parameter *w* is implied with the original parameter without input vector *i*_*t*_. The predicted value is provided for the reparametrized model with the response observed with the original model.

The transformation of parameter has two classes generally. The transformation with expected value and parameter with stability is proposed in this paper. The set of input vector is chosen for the analysis, and expected value is used *w*_1_, *w*_2_,…, *w*_*r*_.

(6) $$\begin{array}{c} wi={\text{fit}}^{*},\;v,t=1,2,\ldots ,r. \end{array}$$

The original parameter *v* is eliminated after transformation of equation. The transformation with the second class is obtained for the parameter estimation.

(7) $$\begin{array}{c} w_{i}=w_{i}^{i}\text{ for }\gamma \neq 0, \end{array}$$

(8) $$\begin{array}{c} w_{i}=\text{ln}\left(u_{i}\right)\text{ for }\gamma =0. \end{array}$$

The transformation parameter has the advantage with constant random variable which enhance the contour form of the surface of response and the possessions of sampling with estimating the least square for the combination of model and data set. When compared to the original model parameterization, one drawback of predictable value alterations is the numerical complication of their formulations [16]. The reparametrized model response function has many converted parameters, and the difficulty of these purposes increases as the number of parameter model increases. Another disadvantage is that it is not always possible to solve the equations to create terminologies for the inventive values just in terms of the changed constraints.

### 2.3. Nonlinearity Measure

The sampling properties are assessed using the nonlinearity measure for the estimation of least square. The asymptotic bias is included for the calculation of the bias and the intrinsic curvature measures and effect of parameter curvature. The model specified for the identification of the bias measure and individual parameter estimation is related to parameter transformation.

The derivate matrix element is given as

(9) $$\begin{array}{c} V^{\prime }=\partial f\left(i_{t},\hat{w}\right)dw_{i}. \end{array}$$

The second derivative is given in

(10) $$\begin{array}{c} V^{\prime \prime }=\partial^{2}f\left(i_{t},\hat{w}\right)dw_{i}dw_{j}. \end{array}$$

The function of *J* is defined as

(11) $$\begin{array}{c} J=V^{\prime }.V^{\prime \prime }. \end{array}$$

The element of *W* is given as

(12) $$\begin{array}{c} W=\sum_{t}V^{\prime }.V^{\prime \prime }. \end{array}$$

The bias measure with the definition of Hougaard is defined by

(13) $$\begin{array}{c} E\left({\hat{w}}_{i}\right)-w_{i}=-\frac{1}{2}\sigma^{2}\sum W.J, \end{array}$$

where $E\left({\hat{w}}_{i}\right)$ is the expected value of ${\hat{w}}_{i}$.

The estimation of variance is given by

(14) $$\begin{array}{c} \sigma^{2}=\frac{s\left(\hat{w}\right)}{n-p}. \end{array}$$

The nonlinear behavior is indicated using the percentage bias

(15) $$\begin{array}{c} B\%=\frac{100\left(E\left({\hat{w}}_{i}\right)-w_{i}\right)}{{\hat{w}}_{i}}. \end{array}$$

Each individual parameter with the nonlinear behavior measure is estimated by the asymptotic moment, which is given by

(16) $$\begin{array}{c} E{\left[{\hat{w}}_{i}-E\left({\hat{w}}_{i}\right)\right]}^{3}=-\sigma^{4}\sum_{qrs}J_{iq}.J_{ir}.J_{is}.\left(W_{qrs}+W_{rqs}+W_{sqr}\right). \end{array}$$

The skewness with direct measure is given by

(17) $$\begin{array}{c} Sk=\frac{E{\left[{\hat{w}}_{i}-E\left({\hat{w}}_{i}\right)\right]}^{3}}{{\left(\sigma^{4}g_{i}\right)}^{3/2}}, \end{array}$$

where *g*_*i*_ is nonlinearity indicator.

The shape of the locus solution is refereed using the intrinsic curvature, which is described using

(18) $$\begin{array}{c} f\left(w\right)=\left[f\left(i_{1},w\right),\ldots ,f\left(i_{1},w\right)\right]. \end{array}$$

The curvature measure for the element *V*′ and *V*′′ is assumed by the standard radius of the element:

(19) $$\begin{array}{c} A=\left[Q^{\prime }\right]\left[L^{\prime }V^{\prime \prime }L\right]. \end{array}$$

The equation is called square bracket multiplication. The decomposition of *V*′ is given by(19).

(20) $$\begin{array}{c} V^{\prime }=qR. \end{array}$$

The parameter effect of the array accelerated in the curvature is denoted as *A*_*PE*_, and intrinsic acceleration is denoted as *A*_*IN*_. The maximum curvature of parameter effect and intrinsic culture is defined by

(21) $$\begin{array}{c} \text{intrinsic curvature}=\text{max}\left\|u^{\prime }A_{IN}u\right\|, \end{array}$$

(22) $$\begin{array}{c} \text{parameter effect curvature}=\text{max}\left\|u^{\prime }A_{PE}u\right\|, \end{array}$$

where u is unit vector, and norm of vector is represented using the vertical bars.

## 3. Methodology

### 3.1. Hydraulic Model

The 1-D modeling technique for flood propagation is still frequently utilized in engineering practice because of its ease of implementation, low processing time, and good real-time operational efficiency. Because of the long channel length, limited floodplain, and low sinuosity, as well as the fact that our flood forecasting is primarily focused on discharge and stage at cross sections rather than flood inundation, a 1-D modeling approach is used to achieve real-time flood forecasting efficiently and adequately in this study. As a result, the hydraulic model used here is the basic 1-D unstable open-channel flow model, which is described using the Saint Venant formulation as follows:

(23) $$\begin{array}{c} \frac{\partial A}{\partial T}+\frac{\partial D}{\partial L}=d_{L}, \end{array}$$

(24) $$\begin{array}{c} \frac{\partial D}{\partial T}+\frac{\partial }{\partial L}\left(\frac{\alpha D^{2}}{A}\right)+gA\frac{\partial S}{\partial L}+g\frac{D\left|D\right|n^{2}}{AR^{4/3}}=0. \end{array}$$

*T* is time, *L* is distance of space along the channel, *D* is discharge, *S* is stage, *α* is correction factor of momentum, *A* is area of cross section, *R* is hydraulic radius, *d*_*L*_ is lateral discharge, and *n* is coefficient of Manning's roughness.

These are hyperbolic partial differential equations that do not have an analytical solution. With sufficient beginning and boundary conditions, the equations may be numerically solved using the four-point implicit finite-difference approach. The four-point implicit finite-difference technique has the advantages of being unconditionally stable, exceedingly robust, and quick to compute. The scheme's fundamental flaw is that it does not operate well when the river topography is complex, which is not the situation with this project. Inflow discharges occur at the upstream and lateral boundary conditions, whereas outflow phases occur at the downstream boundary circumstances.

It is important to mention that the aforementioned fundamental governing equations' form implies the assumption of uniform velocity distribution over the whole cross section. When out-of-bank flow occurs in compound channels, the difference in water depth and flow resistance between the main channel and the floodplains causes a flow velocity differential between these subsections. To mimic the various flow characteristics in subsections, greater momentum correction factors, transverse changes in Manning's roughness coefficient, and momentum transfer between main channel and floodplains should be addressed in the governing equations. The cross sections are simplified as simple channels, eliminating the impact of floodplains, because the geometry of the cross sections in the study channel and the floodplains are small or not formed in the mountain valleys. As a result, the 1-D hydraulic model's essential governing equations maintain the traditional form, with an overall Manning's roughness coefficient for each cross section. Another benefit of employing an overall Manning's roughness coefficient in hydraulic models is the simplicity with which it may be updated during the assimilation process.

The collective resistance to open-channel flow is represented by Manning's roughness coefficient. Its value is influenced by flow circumstances such as the area of submerged vegetation and flow turbulence intensity, as well as physical parameters such as bed geology and cross-sectional geometry. The overall Manning's roughness coefficient for a cross section is a composite number that includes all elements' contributions to flow resistance and may be calibrated using hydrological measurements. The Manning's roughness coefficients in the flood-forecasting model should be permitted to change both geographically and temporally, given the longitudinal fluctuation of a channel's physical properties and the unstable flooding flow. A method for segmenting a river reach is provided, with each segment defined by two adjacent hydrological stations and a series of spatially unique cross sections. Although the cross sections within a river segment have the same roughness coefficient, each river segment has its unique Manning's roughness coefficient. The Manning's roughness coefficient values are calibrated using a trial-and-error technique based on historical flow data and then updated using real-time stage measurements from hydrological stations to represent the unstable flood's instantaneous changes in flow resistance.

### 3.2. Bayesian Framework

A competent hydrologist is typically aware of the potential values, or range of values, of model parameters before any observed data are collected. This knowledge might be based on personal preferences, theoretical considerations, or other factors or an objective assessment of indirect data, such as model parameter regressions on catchment feature. To give reliable parameter estimates, Bayesian estimation combines pre-existing knowledge with observed data (sample information). The unknown parameter vector *u* is regarded as a random variable and distributed using a probability density function in this approach. The information of posterior probability density function is used to combine the Bayes theorem.

(25) $$\begin{array}{c} f\left(\theta |O\right)=cL\left(O|\theta \right)f\left(\theta \right). \end{array}$$

*O* is the vector of observation, the likelihood function *L*(*O|θ*) which contains the information sample. The prior information is given as *f*(*θ*), and *c* is the constant of normalization which need a density function *f*(*θ|O*). By locating the typical value of posterior distribution, various estimations of *θ* are analyzed.

Because the exact form of *f*(*θ|O*) is too complex to calculate, a strict request of Bayesian approximation is not practicable for the types of replicas and sample sizes under consideration.

### 3.3. Bayes Theorem

A Bayesian technique to hydrological modeling offers a persuasive mechanism for integrating precise parameter uncertainty estimations with readily available expert knowledge. Bayesian statistics recognizes preceding data based on historic data or knowledge form expert, as well as data composed via investigation and opinion, as two types of data for knowledge about unidentified parameters. The unknown vector parameter *θ* is modeled as a random variable with a probability density function that represents uncertainty. This function of density is designated *P*(*θ*) and detains all known knowledge about the parameters prior to gathering data. The density is represented by *P* (*θ*|*Q*) prior to gathering data *Q*. Prior and posterior densities are the terms used to describe these densities. Prior densities are less concentrated than posterior densities, which are analyzed using the knowledge about the θ is acquired through the process of collecting data is reflector with a decrease in uncertainty. By using Bayes theorem, the process of updating posterior distribution from prior distribution is given by

(26) $$\begin{array}{c} P\left(\theta |Q\right)=\frac{P\left(\theta |Q\right)P\left(\theta \right)}{P\left(Q\right)}. \end{array}$$

The proportionality constant (27) is represented by *P*(*Q*) which is required for

(27) $$\begin{array}{c} \int P\left(\theta |Q\right)d\theta =1. \end{array}$$

The likelihood function of the sample is given as *P*(*θ|Q*)

(28) $$\begin{array}{c} P\left(\theta |Q\right)=\underset{l,j}{\prod }2\pi \sigma_{l}^{2}\text{exp}\left(-\frac{\left[Q-x\left(r;\theta \right)\right]}{2\sigma_{l}^{2}}\right). \end{array}$$

The information available about the vector parameter *θ* is contained in the posterior distribution. The posterior probability distribution is summarized by reducing the interference of the statistical Bayesian.

### 3.4. Markov Chain Monte Carlo (MCMC) Method

It is difficult to describe the subsequent dispersal by direct computation for even modestly complicated issues with realistic prior belief conditions. MCMC techniques provide an alternate way. This is a method for creating samples from high-dimensional distributions, such as the posterior distribution *P*(OIQ). The goal is to obtain a big enough sample to correctly characterize any desired aspect of the posterior.

The basic concept is to begin with a random beginning value *o* and produce a sequence of reliant on parameter values {*θ*^*t*^ : *t*=1,2,…} from a well-prepared Markov chain: in other words, a random walk across the parameter state space. To create this arbitrary walk, need to design a transition density function that describes the move *θ*^*t*^⟶*θ*^*t*+1^ in such a way that the chain's observed values converge in distribution to the posterior. Before the chain's limiting distribution is achieved, there is usually an unstable transient phase. The chain is considered to have congregated in dispersal after this phase. The iterations collected before convergence are eliminated, leaving a reliant on sample drawn from the subsequent. If the chain is protracted after convergence to gather a large enough sample, the replicated standards of the chain may be utilized to summarize the characteristics of the posterior distribution.

The most commonly used Markov Chain Monte Carlo method is metropolis hasting algorithm. The transition form of *θ*′=*θ* to *θ*^*t*+1^=*θ*′ is described using the probability function. *P*(*θ*, *θ*′) is constructed using metropolis hasting algorithm, which is given as follows:

- The candidate value *θ*′ is generated from the probability distribution *Q*(*θ*, *θ*′) for essentially arbitrary
- The probability *R*(*θ*, *θ*′) is accepted and moved the value to *θ*^*t*+1^=*θ*′
- With the probability 1-*R*(*θ*, *θ*′), *θ*=*θ*^*t*+1^ is set, if the move is rejected

The transition probability with Markov chain is given by

(29) $$\begin{array}{c} P\left(\theta ,\theta^{\prime }\right)=Q\left(\theta ,\theta^{\prime }\right).R\left(\theta ,\theta^{\prime }\right),\\ P\left(\theta ,\theta^{\prime \prime }\right)=\int_{\theta^{\prime \prime }}Q\left(\theta ,\theta^{\prime \prime }\right)\left[1-R\left(\theta ,\theta^{\prime \prime }\right)\right]d\theta^{\prime \prime },\\ P\left(\theta ,\theta^{\prime \prime }\right)=1-\int_{\theta^{\prime \prime }}Q\left(\theta ,\theta^{\prime \prime }\right)\left[1-R\left(\theta ,\theta^{\prime \prime }\right)\right]d\theta^{\prime \prime }. \end{array}$$

Let *R*(*θ*, *θ*′) be defined for the convenience of *P*(*θ|Q*)=*π*(*θ*)

(30) $$\begin{array}{c} R\left(\theta ,\theta^{\prime }\right)=\left\{\begin{array}{c} \min\left(\frac{\pi \left(\theta^{\prime }\right)Q\left(\theta^{\prime },\theta \right)}{\pi \left(\theta \right)Q\left(\theta ,\theta^{\prime }\right)},\;1\right),\;\text{if }\pi \left(\theta \right)Q\left(\theta ,\theta^{\prime }\right)>0,\\ 1,\;\text{if }\pi \left(\theta \right)Q\left(\theta ,\theta^{\prime }\right)=0. \end{array}\right. \end{array}$$

Then,

(31) $$\begin{array}{c} \pi \left(\theta \right)Q\left(\theta ,\theta^{\prime }\right)=\pi \left(\theta^{\prime }\right)Q\left(\theta^{\prime },\theta \right). \end{array}$$

The reversibility condition is defined by the above equation which has the limited distribution for *π*(*θ*) with sufficient condition for the provided chain *Q*(*θ*, *θ*′) irreducibly chosen.

The probability with univariant density function is given by

(32) $$\begin{array}{c} R_{t}\left(\theta ,\theta^{*}\right)=\text{min}\left(\frac{\pi \left(\theta^{*}\right)Q\left(\theta^{*},\theta \right)}{\pi \left(\theta \right)Q\left(\theta ,\theta^{*}\right)},\;1\right). \end{array}$$

### 3.5. Particle Filter

The state variables and constraints are characterized by likelihood distributions that provide for their uncertainties to account for the state-space formulation's stochastic character. Because the state-space preparation is recursive, it may be used to absorb experimental data as it becomes accessible in a sequential manner. From time t-1 through *t*, the conditional probability recursive equation may be used to represent the association among observational data and model outputs. The conditional probability is given

(33) $$\begin{array}{c} P\left(u_{t},\theta_{t}/v_{t}\right)=\frac{P\left(v_{t}/u_{t},\theta_{t}\right)}{P\left(v_{t}/v_{t-1}\right)}P\left(u_{t},\theta_{t}/v_{t}\right), \end{array}$$

*P*(*u*_*t*_, *θ*_*t*_/*v*_*t*_) is probability distribution of posterior model. *P*(*u*_*t*_, *θ*_*t*_/*v*_*t*−1_) is probability distribution of prior mode at time *t*, which is distributed as time *t* − 1. *P*(*v*_*t*_/*u*_*t*_, *θ*_*t*_) is the likelihood function. *P*(*v*_*t*_/*v*_*t*−1_) is normalized constant.

Analytic functional formulations of the likelihood distributions of variables and constraints for complex systems are difficult to come by. To approximate the distributions, the PF uses an ensemble particle. When the number of particles is high enough, particles created via Monte Carlo simulation can reasonably resemble real probability densities. The likelihood distribution of posterior state variable model and constraints at time *t* may be estimated using the ensemble of particles, as follows:

(34) $$\begin{array}{c} P\left(u_{t},\frac{\theta_{t}}{v_{t}}\right)=\sum_{i=1}^{N}w_{t}\delta \left(u_{t}-u_{t}^{\prime },\theta_{t}-\theta_{t}^{\prime }\right). \end{array}$$

*N* is number of particle, *w*_*t*_ is weight based on *i*th particle, and *δ*(.) is Dirac delta function.

The two phases of recursive model approaches based on the particle filter are model forecast and filter correction:

(1) *Model Forecast Phase*. For each particle, from time *t* − 1 until time *t*, each set of state variables and parameters in the dynamic nonlinear model is performed. Each particle represents the prior probability distribution at time *t* following integration, as well as the subsequent probability distribution of a set of state variable quantity and parameter model at time *t* − 1 prior to combination.
(2) *Filter Correction Phase*. Filter correction consists of three steps such as likelihood computation, resampling, and agitation. The goal of probability computation is to inform each particle's weight depending on available data. The Gaussian likelihood function for calculating the likelihood value of each particle is given by (33).
  (35) $$\begin{array}{c} w_{t}=\frac{1}{\sqrt{2\pi \sigma }}\text{exp}\left(-\frac{{\left(u_{t}-v_{t}\right)}^{2}}{2\sigma^{2}}\right), \end{array}$$
where *u*_*t*_ is prior state variable at time *t*, *v*_*t*_ is state variable observation at time *t*, and *σ*^2^ is standard deviation.

By normalizing the likelihood value, the particle weight is determined by summing all the likelihood value of the particle.

(36) $$\begin{array}{c} W_{t}=\frac{w_{t}^{i}}{\sum_{i=1}^{N}w_{t}^{i}}, \end{array}$$

where *W*_*t*_ is normalized weight.

The random noise of the perturbed particles incorporated to the parameter model is given by

(37) $$\begin{array}{c} \emptyset_{t+1}=\emptyset_{t}+\epsilon , \end{array}$$

where *ϵ* is Gaussian distributed with random noise.

After determining the normalized weights, resampling is done. Particle degeneracy occurs when the majority of particles have insignificant weights and just a few elements are efficient in the strainer. It is necessary to resample the particles in order to remove the small weighted elements and develop auspicious new elements based on the high weighted elements in order to decrease the meaningless calculations and more precisely predict the current time step's condition.

To keep particle variety, perturbation is used. Though resampling can reduce immorality, the variety of elements may suffer as a result of sample impoverishment, in which some particular elements with great weights are reproduced multiple times. It is a waste of time to keep running the same model with the same elements. The disturbed sub-divisions can move about in space in a stochastic way to follow their development over time. By including random noise in the model parameter, the particles can be disturbed.

### 3.6. Parameter Estimation

The assortment of an objective function that shows quality of fit is required for parameter estimation by comparing calculated and experimental hydrographs. This function must be appropriate for the model's intended application. Reproduction of experimental flood hydrograph peaks is regarded to be extremely important in many flood investigations. As a result, the suggested goal function focuses on the fitting of calculated and pragmatic hydrograph peaks.

### 3.7. Objective Function

Consider a rainfall runoff data issue with a *p* parameter model and *n* discrete storm occurrences. Assume the observed and calculated peak discharges are correlated as follows:

(38) $$\begin{array}{c} O=h\left(\theta \right)+u, \end{array}$$

where *O* is the observed vector peak discharge, *h*(*θ*) is the computed vector of the peak discharge, *θ* is the vector parameter, and *R* is the covariance and zero mean with Gaussian error. The prior information is pooled with the sample information and the parameter vector with prior mean and covariance *M*:

- First, when just the prior mean and standard deviation of *u* are known, the supposition of a Gaussian prior probability density function contributes the least volume of unnecessary data to the valuation issue.
- Second, the Gaussian assumption is not dangerous in repetition and its influence is smaller for large *n*.
- Third, the section that contains the inverse of *M* works as a constraint on the components of *u* m. However, when additional floods are included in the sample data, the influence reduces.
- Fourth, timing errors caused by the rainfall with poor synchronization and runoff time series have little effect on parameter estimations.

### 3.8. Algorithm of Optimization

Minimization necessitates the employment of a nonlinear, unconstrained optimization technique for representations in which *h*(*u*) is a function of nonlinear *u*. For the sort of issue at hand, the algorithms provided based on the Gauss technique are preferable than flexible metric approaches and direct search methods. As a result, the aforementioned optimization issue was solved using the Gauss–Marquardt method. The best aspects of the steepest descent and Newton techniques are combined in this method. The basic repetition in these Gauss–Marquardt methods is

(39) $$\begin{array}{c} x_{i-1}=x_{i}-AG_{i}. \end{array}$$

The matrix A is defined as

(40) $$\begin{array}{c} A={\left(M+\mu B\right)}^{-1}. \end{array}$$

Gauss approximation represented *M*, and *μ* is the chosen scalar with positive derivatives. The optimization of gradient vector is described as

(41) $$\begin{array}{c} G_{i}=-2L^{-1}\left(\theta_{0}-\theta \right)-2DR\left(x-h\left(\theta \right)\right). \end{array}$$

### 3.9. Estimation of Pooling

NFEMs explain a strategy for parameter estimate across storm occurrences. This approach includes the concept that pooling is justifiable, as well as the common parameter model (CPM) with parameter vector. ts is a vector holding storm-specific parameters, whereas *t* is a vector containing parameters that are common to all storm occurrences in the CPM. If pooling is not allowed, a more generic data model is needed.

The competing model is selected by specifying the beliefs prior to the parameter, and common parameter model with prior odds is specified for easy processing.

MC = common parameter model is true and *M*_G_ = general parameter model is true. The odd prior is represented as *P*(*M*_*C*_)/*P*(*M*_*G*_). The odd posterior is represented as *M*_*C*_ and *M*_*G*_ as given in

(42) $$\begin{array}{c} \frac{P\left(M_{C}/Q\right)}{P\left(M_{G}/Q\right)}=\frac{P\left(Q/M_{C}\right)}{P\left(Q/M_{G}\right)}.\frac{P\left(M_{C}\right)}{P\left(M_{G}\right)}. \end{array}$$

The Bayes quantity factor for representing the common and general parameter is given as *B*_*CG*_, which is used to select the parameter among the hydrological model. The marginal probability of *B*_*CG*_ is denoted as

(43) $$\begin{array}{c} P\left(\frac{Q}{M}\right)=\int_{\theta }P\left(\frac{Q}{M},\theta \right)P\left(\theta \right)\text{d}\theta . \end{array}$$

The equation is related to Monte Carlo average and concentrated with posterior mode distribution. The harmonic mean estimation is given as (42).

(44) $$\begin{array}{c} P\left(\frac{Q}{M}\right)={\left[N^{-1}{\sum P\left(\frac{Q}{\theta }\right)}^{-1}\right]}^{-1}.\; \end{array}$$

The large fluctuation in the likelihood value is estimated occasionally. The sophisticated technique is applied for potentially generating the finer answer.

### 3.10. Uncertainty in Flood Estimation

There are two sorts of prediction failures in flood forecasting: (a) the system may fail to give a warning for a flood event, and (b) it may issue a warning for an event that does not materialize due to an error of commission. A flood in the first situation might result in the loss of lives, infrastructure, and property [17]. People may lose faith in the forecast in the second situation and fail to respond to the following warning. To reduce the chances of any form of failure, it is vital to analyze and communicate forecasting mistakes and uncertainties.

### 3.11. Source of Uncertainty and Error

In flood forecasting, there are several sources of uncertainty such as input data uncertainty, model uncertainty, and model parameter uncertainty. The several causes of mistakes in the flood warning process have been recognized, and general methodologies for analyzing uncertainty have been offered. Precipitation and anticipated is a crucial meteorological input in forecasting flood. Forecasted precipitation is usually produced from numerical weather prediction's quantitative precipitation predictions [18]. The numerical weather prediction grid size may be a substantial source of mistake in rainfall forecasting, which is exacerbated by the grids' positional error. Even observed precipitation is subject to considerable uncertainty. Rain gauges only cover a small region, and there might be significant gaps between them, resulting in huge precipitation inaccuracies, especially in mountainous places. Weather radars may cover wide regions but cannot directly monitor rainfall, and conversion from reflectance to rainfall might be problematic. Flood-producing storm events often occur on small scales, and gridded remotely sensed products may not catch them.

Besides from precipitation, a variety of additional mistakes and uncertainties can be significant. When the models are applied to individual storm occurrences, for example, inaccuracies connected with the beginning circumstances, e.g., soil moisture, are particularly critical. Furthermore, any model update or downscaling, as well as infrastructure activities, might introduce mistakes and uncertainty. With the usage of rating curves, errors might be introduced. Flood forecasts are usually presented as level (gauge data), but hydrological models usually compute discharge. To convert computed fluxes to water levels, a rating curve is employed. Rating curves are often created using a small number of discharge observations that may not cover severe flood occurrences, leaving enough opportunity for error. Furthermore, the gauge observations may have uncertainties. Furthermore, erroneous or missing data, human processing mistakes, or unforeseen actions can all contribute to operational uncertainty in flood forecasting [19].

Finally, model structural errors, parameter mistakes, and spatial discretization errors can all result in large inaccuracy when forecasting models misrepresent hydrologic processes. Model structure arises from the simplification of intricate catchment physics into models of physical processes. More data are not going to fix these systemic issues. There may be a lot of uncertainty in model parameters, but this usually goes away when more recorded runoff data become available and are utilized to change the model parameters. The size of the research region, the diversity of its characteristics, the number of sub-divisions of the area, and the data resolution all impact parameter uncertainty. The geographical representation of the catchment, of which there are three popular techniques such as sub-catchments, rectangular grid model, and response units such as SWAT model, is a significant inaccuracy related to forecasting models.

### 3.12. Uncertainty Quantification

Quantifying flood-forecasting predictive uncertainty is critical for conveying flood danger and minimizing uncertainties. Uncertainty analysis is one of the most challenging components of flood forecasting since a thorough definition of the cascade of uncertainties from input to warning is a time-consuming and computer-intensive approach. There are several methods for quantifying the uncertainty of specific forecasting components. One method is to modify the expected rainfall and model parameters to test the model's sensitivity to a believable range of inputs. Because parameter model may be dependent on previous standardization and knowledge, there is a lot of uncertainty in the early stages of forecasting. Improved model calibration raises parameter confidence and reduces uncertainty as the event progresses and more observable data become accessible. The relevance of observed data is underscored by the findings, which show that additional stage data are more useful than parameterization of flood models in lowering barrage depths and range uncertainty [20].

The predictive uncertainty paradigm depicts the likelihood of a forthcoming value of a forecast, such as water level and discharge, occurring. This likelihood is based on all available knowledge about the future value, which is often supplied via forecast modeling. The predictive uncertainty technique has the benefit of quantifying uncertainty in terms of a likelihood distribution.

The impacts of a wide variety of forecast uncertainties can be included into the abovementioned ensemble prediction systems (EPSs), yielding a probabilistic forecast with directly expressed uncertainty. To indicate uncertainty, individual models within the ensemble may have different assumptions regarding beginning circumstances, boundary conditions, model parameterization, model structure, or any combination of these. Ensembles can be assembled in a variety of ways. The most common technique involves feeding numerical weather predictions from an ensemble prediction system into hydrological models to obtain hybrid ensemble prediction system-based forecasts. This technology was used to create the European Flood Awareness System [21].

Within the ensemble, some ensembles utilize many models or the same model with various physical parameterization methodologies. Ensemble forecasting strategies include using climatology and starting circumstances to build an ensemble, leveraging error distributions derived from previous hydrological forecasts to improve current predictions, and handling spatial uncertainty in rainfall forecasting. Rather than a single deterministic forecast, a set of projections may be used to quantify and explain the uncertainty surrounding the flood event [22].

Hybrid ensemble prediction methods, while widely used, are not without their drawbacks. In many circumstances, hybrid ensemble prediction systems must analyze enormous volumes of data produced by collaborative models that are not always available or computationally practical. Understanding how to base flood warning decisions on probabilistic projections is difficult. Emergency response agencies, for example, may be perplexed by probabilistic estimates and react with fear or apathy. Furthermore, efforts must be made to describe and convey the elements that influence the accuracy of forecasts based on hybrid ensemble prediction systems. Probabilistic hybrid rainfall forecasts influence the performance of hydrological predictions. An evaluation of hydrological performance is based on estimating the bias of deterministic and collaborative hydrological estimates impacting the catchment outlet discharge threshold. To evaluate ensemble predictions, a peak-box technique is used. The extent and effectiveness of peak discharges are the emphasis of this method [23].

Furthermore, decision-making based on probabilistic predictions provided by hybrid ensemble prediction systems needs visualization tools and user-focused ensemble forecast assessment. Despite the significance of prediction visualization and communication approaches, individuals who receive forecasts see, understand, and act on them. If predictions are to be effective for flood control, developing visualization tools and estimating the products for expressing indecision is a critical challenge.

## 4. Results and Discussion

The study analyzes the application of nonlinear flood event in the Harvey River catchment with the description of data based on rainfall, model, and prior information available. The procedure of estimation is checked based on the several assumptions. The various stages of the flood event are analyzed based on the parameter estimation using Bayesian theorem. The information about the region of study is given in Table 1.

**Figure 1:**
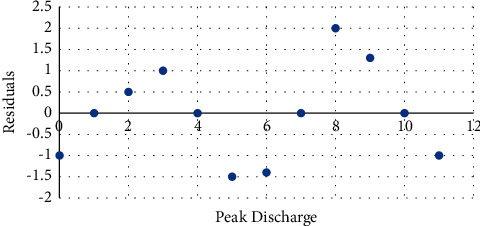
Least square with the standardized residual plot.

The error in least square is assumed with the residual plot, and the correlation in the serial is tested. The error variance with constant value is sampled using the Bayesian parameter and pooling diagnosis, which is given in Figure 1.

**Figure 2:**
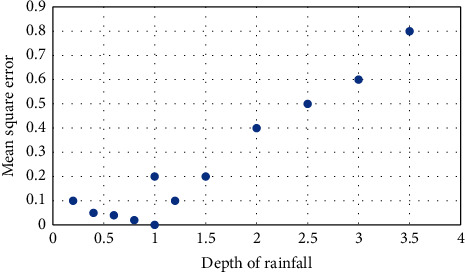
Quadratic relation.

The relationship among the mean depth of the rainfall and error variance is revealed using river catchment. The data set of each element in the matrix is established in Figure 2. The variance of the peak discharge is observed using the regression model.

**Figure 3:**
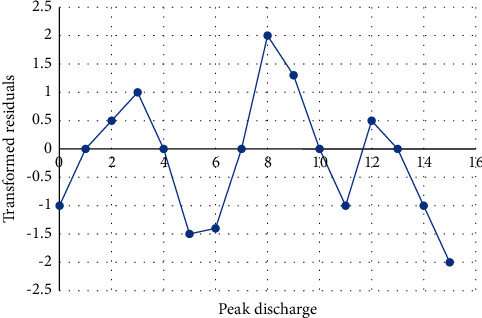
Plot of residual for weighted least square.

The transformed residual plot is shown in Figure 3, which indicated the variability of the residual transformed and transformed dependent on the weighted least square function.

**Figure 4:**
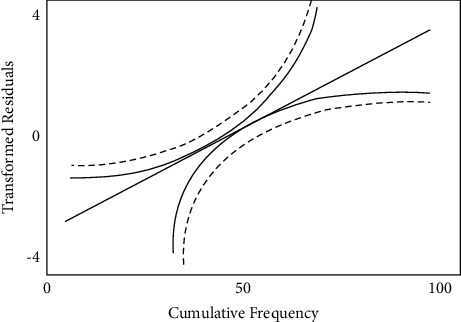
Plot of normal probability.

The plot of normal probability is shown in Figure 4, which is observed for transmuted residuals for Harvey River samples. The curve denotes the Gaussian distribution and critical value of the statistical analysis. The altered residuals are carefully conformed to the Gaussian distribution function.

The result of maximum a posterior is obtained in Table 2 for Harvey River sample. The estimation of MAP is precise which is prior to the corresponding mean. The utilization of MAP estimation enhances the precision improvement.

## 5. Conclusion

A Bayesian technique is developed for estimation of parameter in flood event with nonlinear models in this research. The approach is fairly broad, and it may be used to convert any perfect into a nonlinear regression framework. The use of a maximum a posteriori (MAP) approach for estimation of parameter in flood event models is discussed, with the goal of improving the dependability of design flood hydrographs. The suggested technique combines previous information on the model parameters as well as information of the mistake structures of the input data into the approximation process. The matrix of posterior covariance is measured using the calculated parameters' accuracy.

## Figures and Tables

**Table 1:** Prior information about the Harvey River sample.

Area	148

Relief	Low to moderate
Length of record available	13
Measurement of maximum discharge	20
Recording the maximum number of rain gauge	4
Number of storm	35

**Table 2:** Model parameter with mean and variance.

Parameter	Mean	Variance
Ordinary least square	0.038	0.054
Weighted least square	0.034	0.058
Prior parameter	0.13	0.62
Maximum a posterior	0.095	0.064

## Data Availability

The data used to support the findings of this study are available from the corresponding author upon request.
